# Sharing Caregiving for Older Adults with Dementia at Home: Primary Caregivers’ Perspectives of Informal Caregiving Networks

**DOI:** 10.1002/alz70858_098247

**Published:** 2025-12-25

**Authors:** Mi‐Kyung M Song, Annette DeVito Dabbs, Mary Beth Happ, Byan Michael Wiley, Haerim Lee, Waliah Saif, Jiyoon Jang, Naziya Noorani

**Affiliations:** ^1^ Emory University, Atlanta, GA, USA; ^2^ University of Pittsburgh School of Nursing, Pittsburgh, PA, USA; ^3^ The Ohio State University, College of Nursing, Columbus, OH, USA; ^4^ Oregon Health Science University, Portland, OR, USA; ^5^ Emory University, School of Nursing, Atlanta, GA, USA

## Abstract

**Background:**

Caregiving for older persons with dementia (PWD) commonly involves more than a single caregiver, i.e., a network of caregivers. Yet, research examining the experiences of shared caregiving among caregiving network members to meet PWD care needs has been limited.

**Method:**

We used maximum variation sampling to enroll 46 primary caregivers of community‐dwelling PWD who had diverse characteristics, e.g., gender, race/ethnicity, relationship to PWD, caregiving years, network size (total number of informal caregivers). One‐on‐one interviews included questions about the primary caregiver's experiences in sharing caregiving tasks with network members, such as division of tasks, coordination, and communication. Interviews were recorded, transcribed, and imported to a software package to organize data for analysis for the purpose of qualitative description. Using the scripted questions as a scaffold, two researchers independently coded each transcript, then reached consensus on the final codes, and clustered them under themes.

**Result:**

Participants were 60 years old on average, mostly women (83%), non‐white (60%), daughters of the PWD (61%), and provided care for 6 years on average. Most networks (61%) were comprised of 3 or 4 (range, 2‐9), primarily immediate family members. Participants reported benefits of sharing caregiving, including reducing burden, providing respite, bringing family closer, providing the opportunity to bounce off ideas with each other, keeping caregiving within the family, promoting PWD's well‐being, and being able to care for PWD at home. Reported challenges of sharing caregiving included different caregiving styles and opinions among members, getting everyone on the same page, and coordinating schedules. In most cases, the primary caregiver was responsible for coordination and reported that they had the final say in decision‐making. Division of tasks was based on member's availability and ability or skill matching, relationship with PWD, or network size. Participants attempted to prevent or resolve communication breakdowns by keeping network members informed and settling disagreements through open discussion and compromise. However, some used avoidance strategies to circumvent confrontation for fear of losing network members.

**Conclusion:**

Findings reveal the overlooked social contexts in which caregiving occurs, including the interactions among multiple caregivers, and suggest potential areas of caregiving burden and interventions.

